# Bezold-Jarisch Reflex Presenting With Bradypnea, Bradycardia, and Hypotension Following Combined Spinal Epidural Prior to Cesarean Section: A Case Report

**DOI:** 10.7759/cureus.53643

**Published:** 2024-02-05

**Authors:** Colin Kirsch, Areen Badwal, Romain Rabany, Julia Shabanian, Carla L Dormer

**Affiliations:** 1 Anesthesiology, Creighton University School of Medicine, Phoenix, USA; 2 Psychiatry, Creighton University School of Medicine, Phoenix, USA; 3 Anesthesiology, Arizona Health Education Alliance, Creighton University, Phoenix, USA; 4 Anesthesiology, Valleywise Health Medical Center, Phoenix, USA; 5 Anesthesiology, District Medical Group, Phoenix, USA

**Keywords:** anesthesia, intraoperative, cardiac mechanoreceptors, emergency cesarean delivery, neuraxial anesthesia, bezold-jarisch reflex (bjr)

## Abstract

The Bezold-Jarisch reflex (BJR) is an inhibitory reflex characterized by bradycardia, hypotension, and apnea originating from ventricular mechanoreceptors. BJR is an uncommon but serious complication of neuraxial anesthesia. We present a case of a 33-year-old female undergoing combined spinal-epidural anesthesia prior to cesarean delivery who developed profound BJR, resulting in emergent actions. Within minutes of injection, she became severely bradycardic (HR: 17 bpm) and hypotensive (SBP: 30s mmHg) with bradypnea (RR: 6/min) and was treated with epinephrine. Fetal bradycardia prompted emergency cesarean section. Following delivery, the patient developed ventricular tachycardia, which was treated with intravenous fluids and cardiac monitoring. Both patient and neonate were discharged in stable condition on postoperative day four. This case illustrates the rapid maternal and fetal compromise associated with BJR during neuraxial anesthesia and the need for prompt recognition and treatment. Key steps include stopping anesthesia, intravenous fluid, left-lateral positioning, judicious vasopressors, fetal monitoring, and preparing for emergent delivery.

## Introduction

The Bezold-Jarisch reflex (BJR) is an inhibitory reflex characterized by the sudden onset of bradycardia, hypotension, and apnea originating from intracardiac mechanoreceptors located in the inferoposterior wall of the left ventricle [1]. These receptors, when stimulated, elicit an increase in efferent vagal activity and suppression of sympathetic outflow [2]. BJR is attributed to factors that reduce ventricular volume or increase myocardial contractility, activating the mechanoreceptors [1].

BJR has been reported following various triggers, including upright posture, Valsalva maneuver, inferior wall myocardial ischemia or infarction, therapeutic use of drugs with negative inotropic effects, and neuraxial anesthesia [3]. Of these, neuraxial anesthesia, often used in the obstetric population for analgesia cesarean delivery, poses a particular risk. Sympathetic blockade from neuraxial techniques leads to venous pooling, peripheral vasodilation, and reduced ventricular volume. Concurrently, unopposed vagal tone is increased. The combination of decreased ventricular wall stretch and heightened vagal activity can profoundly stimulate the cardiac mechanoreceptors to elicit BJR [2].

Although uncommon, unintended cardiovascular collapse secondary to BJR in an obstetric patient can have devastating fetal and maternal consequences, including emergency cesarean delivery, perinatal hypoxia, myocardial injury, cardiovascular collapse, and even death. Thus, prompt recognition and immediate treatment of BJR are imperative. We present a case of a patient undergoing combined spinal-epidural anesthesia who developed severe BJR resulting in emergent cesarean delivery to illustrate the serious maternal and fetal risks associated with this phenomenon as well as the challenges in management.

This article was previously presented as a poster abstract at the 2023 ASA Annual Meeting on October 15, 2023.

## Case presentation

**Figure 1:**
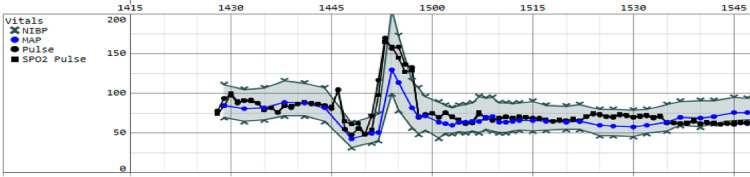
Intraoperative record of patient hemodynamics

A 33-year-old G4P3003 female at 39 weeks and three days gestation presented for an elective repeat cesarean delivery and tubal ligation. Her past medical history was significant for three prior cesarean deliveries with epidurals without complication. Combined spinal-epidural anesthesia was performed in the sitting position using a total spinal dose of 1.4 mL 0.5% bupivacaine, 15 mcg fentanyl, and 0.5 mg morphine. Within four minutes of injection, the patient developed severe bradycardia to 17 beats per minute, hypotension with SBP in the 30s mmHg, and bradypnea with an RR of six per minute, consistent with BJR. The patient lost consciousness at this time. Intravenous epinephrine was administered in four boluses totaling 200 mcg, resolving the bradycardia, hypotension, and bradypnea. However, this was immediately followed by a nine-minute episode of wide complex tachycardia with ST-segment changes concerning ventricular tachycardia before reverting to normal sinus rhythm (Figure 1).

Concurrent with the maternal hemodynamic decline, persistent fetal bradycardia to 70 beats per minute was noted on continuous external fetal monitoring. The nonreassuring fetal HR tracing prompted an emergent cesarean delivery. A healthy infant was delivered with Apgar scores of eight and nine at one and five minutes, respectively. The patient remained hemodynamically stable after delivery, and the rest of the surgery was completed uneventfully.

**Figure 2:**
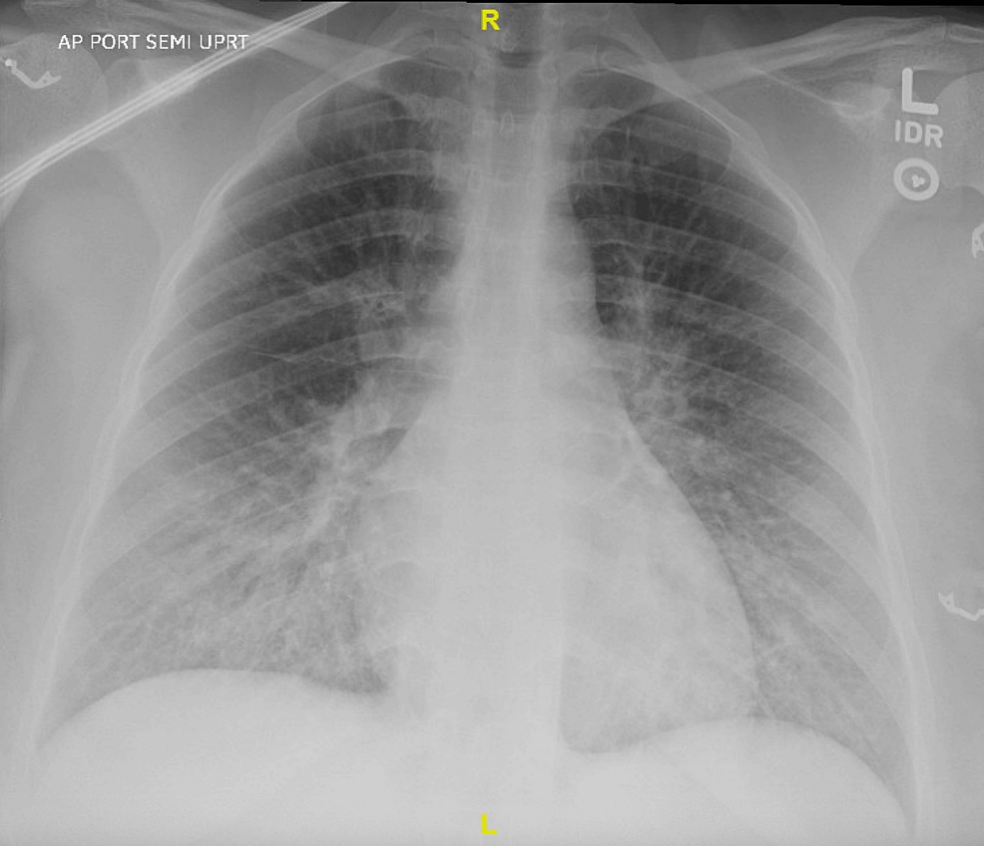
Chest radiograph demonstrating new pulmonary edema The patient's chest X-ray shows worsening diffuse interstitial prominence with peribronchial cuffing predominating at the lung bases.

Postoperatively, laboratory evaluation revealed an elevated troponin level concerning myocardial injury likely resulting from the episode of ventricular tachycardia. Troponins slightly uptrended as high as 5.7 postoperatively, eventually downtrending to 2.21 the following morning. A chest radiograph demonstrated new pulmonary edema, which was attributed to the aggressive 1.4 L of intraoperative intravenous fluid resuscitation given to the patient during the surgery (Figure 2).

The patient had no history of cardiac or pulmonary issues and denied ever having chest pain after the procedure. The cardiology team was consulted, and they recommended conservative treatment, obtaining an echocardiogram, and continuing EKG monitoring. On postoperative night one, the patient required 1-2 L O_2_ from a nasal cannula for dyspnea and O_2_ saturations that dropped to 91% at the lowest. Standard telemetry monitoring showed normal sinus rhythm without ectopy. Both the troponin level and pulmonary edema improved by postoperative day two. On postoperative day three, a transthoracic echocardiogram showed that the left and right ventricles had normal size, wall thickness, and systolic function. The left ventricular ejection fraction was calculated at 61% with normal left ventricular filling patterns. The patient and neonate were discharged home in stable condition on postoperative day four.

## Discussion

This case highlights several key teaching points regarding BJR. First, it sheds light on the sudden and profound cardiovascular collapse that can manifest following this type of anesthesia. The rapid onset of severe bradypnea, bradycardia, and hypotension, accompanied by significant instability, underscores the challenge of implementing proactive measures promptly.

**Figure 3:**
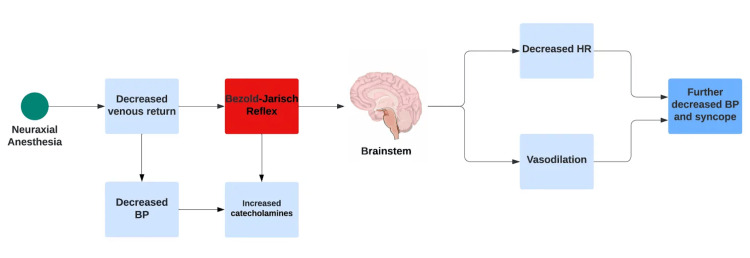
Bezold-Jarisch reflex after receiving neuraxial anesthesia.

Moreover, it demonstrates the diagnostic and treatment challenges intrinsic to BJR. Confirming BJR based on clinical findings alone can prove difficult since the hallmark presentation of bradycardia, hypotension, and bradypnea mirrors that of more common etiologies like vasovagal syncope or incidental high spinal blockade [4]. All three have in common that a stimulus is to blame for the downstream effects of bradypnea, bradycardia, and hypotension, with neuraxial anesthesia being a common cause of BJR (Figure 3). 

Adjunctive monitoring, including ventilation capnography to detect hypopnea or echocardiography to assess ventricular function, may help differentiate BJR from other causes but is often impractical to be used promptly intraoperatively [4]. Consensus guidelines are lacking, given the rarity of BJR episodes and ethical constraints limiting large-scale human studies. Anesthesiologists need to be ready to ventilate a patient experiencing BJR if the apnea does not resolve immediately after raising the blood pressure, and the induction dose of intravenous anesthetic drugs should be reduced significantly if a hypotensive patient needs to be intubated and ventilated emergently. Most evidence regarding pharmacotherapy treatment of BJR stems from animal experiments and anecdotal human case reports, thus clouding risk versus benefit considerations [2]. This ambiguity can handicap clinical decision-making in an already tenuous situation. Current recommendations for first-line treatment of BJR involve stopping the presumed trigger, intravenous fluid boluses to increase preload, and left lateral positioning to minimize vena cava compression [4]. If hemodynamics remain unstable, pharmacologic management including vasopressors like epinephrine, norepinephrine, phenylephrine, or dopamine can be administered to elicit reflex tachycardia and peripheral vasoconstriction. Atropine should be viewed as a third-line option due to the possible deleterious effects of causing ventricular fibrillation [5]. In this case, the patient received multiple boluses of epinephrine with rapid restoration of normal sinus rhythm, HR, and blood pressure. However, she subsequently developed a wide complex tachyarrhythmia, highlighting the risk of over-treatment. As such, medications should be titrated carefully to the minimum dose required for stabilization, and I intravenous fluids should be administered first.

Finally, this case highlights the value of preparedness and teamwork. Preoperative placement of large bore intravenous access, invasive hemodynamic monitors, and emergency medications can help mitigate instability should it arise intraoperatively. BJR has rapid consequences on the fetus when acute maternal cardiovascular instability interrupts uteroplacental perfusion. Fetal bradycardia ensued rapidly after the onset of BJR, necessitating immediate delivery in an unstable mother to prevent further fetal decompensation. Thankfully, the neonate transitioned well and met all milestones postoperatively. Parallel efforts to prevent BJR, including cautious dosing of neuraxial medications and incremental positional changes to avoid precipitous venous pooling, are also prudent. When BJR does occur, outcomes rely heavily on rapid and coordinated care between obstetricians and anesthesiologists to concurrently address maternal stabilization while evaluating fetal tolerance and the need for immediate delivery. Simulation training to establish protocols and choreograph multi-team dynamics may further enhance safety.

## Conclusions

In conclusion, BJR is an uncommon but serious complication associated with neuraxial anesthesia in the obstetric population. Unintentional cardiovascular collapse can endanger both maternal and fetal well-being, necessitating emergent resuscitation and delivery. Challenges exist in promptly recognizing BJR among more common etiologies of intraoperative instability, as well as navigating ambiguous treatment regimens with the potential for unintended harm. Ultimately, preoperative preparedness and vigilance coupled with a coordinated team approach can help mitigate adverse outcomes from this reflex. Increased awareness of BJR through further research and education is paramount.
